# Dynamic 3D genome reorganization during senescence: defining cell states through chromatin

**DOI:** 10.1038/s41418-023-01197-y

**Published:** 2023-08-18

**Authors:** Haitham A. Shaban, Susan M. Gasser

**Affiliations:** 1 Precision Oncology Center, Department of Oncology, Lausanne University Hospital, 1005 Lausanne, Switzerland; 2 Agora Cancer Research Center Lausanne, Rue du Bugnon 25A, 1005 Lausanne, Switzerland; 3 Spectroscopy Department, Institute of Physics Research National Research Centre, Cairo, 33 El-Behouth St., Dokki, Giza, 12311 Egypt; 4 Fondation ISREC, Rue du Bugnon 25A, 1005 Lausanne, Switzerland; 5 Department of Fundamental Microbiology, University of Lausanne, 1015 Lausanne, Switzerland

**Keywords:** Chromatin structure, Diagnostic markers

## Abstract

Cellular senescence, a cell state characterized by growth arrest and insensitivity to growth stimulatory hormones, is accompanied by a massive change in chromatin organization. Senescence can be induced by a range of physiological signals and pathological stresses and was originally thought to be an irreversible state, implicated in normal development, wound healing, tumor suppression and aging. Recently cellular senescence was shown to be reversible in some cases, with exit being triggered by the modulation of the cell’s transcriptional program by the four Yamanaka factors, the suppression of p53 or H3K9me3, PDK1, and/or depletion of AP-1. Coincident with senescence reversal are changes in chromatin organization, most notably the loss of senescence-associated heterochromatin foci (SAHF) found in oncogene-induced senescence. In addition to fixed-cell imaging, chromatin conformation capture and multi-omics have been used to examine chromatin reorganization at different spatial resolutions during senescence. They identify determinants of SAHF formation and other key features that differentiate distinct types of senescence. Not surprisingly, multiple factors, including the time of induction, the type of stress experienced, and the type of cell involved, influence the global reorganization of chromatin in senescence. Here we discuss how changes in the three-dimensional organization of the genome contribute to the regulation of transcription at different stages of senescence. In particular, the distinct contributions of heterochromatin- and lamina-mediated interactions, changes in gene expression, and other cellular control mechanisms are discussed. We propose that high-resolution temporal and spatial analyses of the chromatin landscape during senescence will identify early markers of the different senescence states to help guide clinical diagnosis.

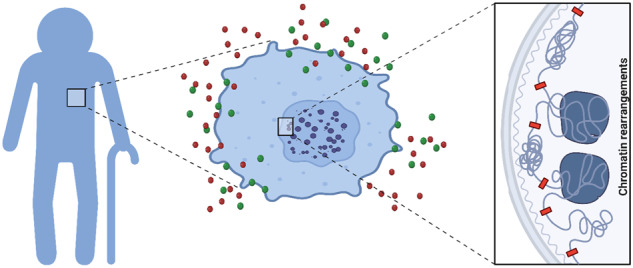

## Introduction

Cellular senescence is a cell fate characterized primarily by an extremely stable proliferative arrest [1]. Its resistance to both proliferative and cell death stimuli means that both apoptotic stimuli and pro-survival pathways are blocked [2]. Senescence thus appears to be an irreversible state of G1-phase cell cycle arrest, with an atypical pattern of secretion (senescence-associated secretory phenotype, or SASP) and internal molecular damage, which itself imbalances cell homeostasis [1, 3]. Senescent cells differ in terms of specific morphological and molecular properties from other non-dividing cell populations (e.g., quiescent cells and/or terminally differentiated cells) [4]. Although senescence has been known as one of the key hallmarks of human aging, it also has beneficial and functional roles in specific physiological and/or pathological processes [5]. Notably, it is a powerful anti-tumor mechanism that can eliminate the proliferation of transformed, cancer-causing cells [6]. A better understanding of senescence entry and exit is thus of major therapeutic relevance.

**Fig. 1:**
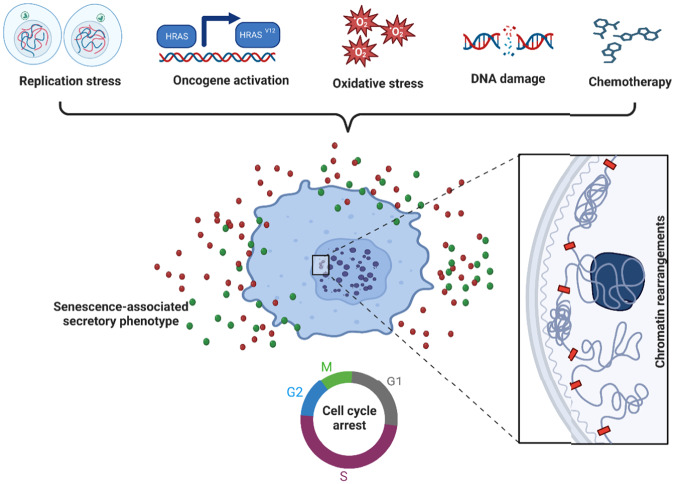
Overview of senescence inducers, and chromatin rearrangement. The senescence program can be activated by different ways (top of the figure in blue) such as: replicative stress (which occurs due to deficiencies in the DNA replication machinery and telomere shortening), oncogene activation, oxidative stress, DNA damage, and chemotherapeutic drugs. The main cellular and molecular effects are shown in red and include an expansion of the lysosomal compartment, metabolic and mitochondrial alterations, accumulation of DNA damage. All these stimuli lead to irreversible arrest of the cell cycle, senescence-associated secretory phenotype (SASP), and rearrangement of the chromatin landscape.

Different types of cellular senescence are classified both by the initial senescence-inducing signals as well as the cell type involved. Exhaustion of a cell’s proliferative capacity followed by irreversible growth arrest induces a phenotype called replicative senescence (RS) [7]. As a result of impaired telomerase activity, telomeres shrink with each cell division cycle, until they trigger a DNA damage checkpoint. Replicative senescence is hypothesized to stop the growth of tumor cells and inhibit tumorigenesis by activating p53 and pRB tumor suppressor proteins [8]. In contrast to the weeks and months required for RS in vitro, oncogene-induced senescence (OIS) is triggered within a relatively short timeframe, that is, within several days, by the overexpression of oncogenes [5, 8]. Like OIS, stress-induced premature senescence (SIPS) is another, distinct type of senescence that can be induced fairly rapidly by oxidative and genotoxic stress, affecting cells from various tissues [9]. One type of SIPS that is clearly distinct from OIS, is the premature senescence brought on by genotoxic medicines, such as the anticancer chemotherapeutic agent bleomycin, which triggers DNA and protein oxidation [10]. Both OIS and SIPS occur regardless of telomere length, yet can correlate with telomere dysfunction. The characteristic features of this state are cell cycle arrest or exit, highly visible chromatin rearrangements, and enhanced secretion or SASP [11] (Fig. 1).

It has been clear in recent years that senescent cells accrue significant chromatin structural changes that coincide with changes in their transcriptional programs [12]. It is still being investigated to what extent these changes in chromatin organization are a cause or an effect of the senescence-linked transcriptional programs. Here, we highlight recent insights as to how cellular senescence affects chromatin structure and transcriptional programs.

## The global reorganization of senescent chromatin

**Fig. 2:**
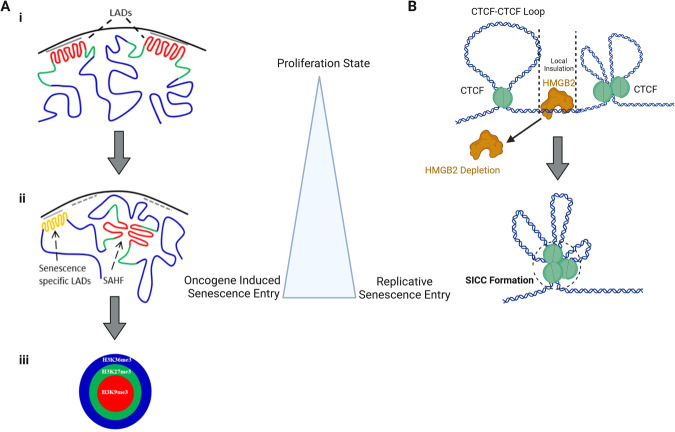
A comparison between nuclear changes upon either oncogene induced senescence or replicative senescence entry. **A** SAHF formation in OIS. i) A growing cell in which LADs (red) are located in the periphery of the nuclear envelope. ii) During cellular senescence, droplets of LADs move to the interior of the nuclear space SAHF formation. LADs consisting of constitutive heterochromatin (red) and are attached to the nuclear lamina (gray). H3K27me3 regions (green) flank LADs. Euchromatic regions are shown in blue, and senescence-specific LADs move towards the periphery (gold). iii) a sketch of the multi-layered architecture of a SAHF, with core and ring of heterochromatin bearing H3K9me3 and H3K27me3, respectively, surrounded by active H3K36me3 chromatin [32]. **B** SICC formation in RS. The binding of HMGB2 protein (gold) to chromatin at TAD boundaries (local insulation) and forms CTCF (green)-CTCF loops. The loss of HMGB2 proteins promote senescence-induced CTCF spatial clustering (SICC) [34].

Accompanying the reduced proliferation of senescent cells is an extensive remodeling of the 3D organization of the genome, generating characteristically protruding blebs and irregularly shaped nuclei [12, 13]. This can largely be attributed to changes at the nuclear envelope, which plays a critical role in ensuring appropriate chromatin distribution and preserving nuclear shape. The nuclear envelope comprises a double membrane that is contiguous with the endoplasmic reticulum, and an underlying meshwork primarily composed of the nuclear lamins. These filament-forming lamin proteins (Lamin A, C, B1, and B2) are the dominant structural components of the nuclear lamina, which is attached to the inner nuclear membrane through transmembrane anchors and nuclear pores (Fig. 2A). The failure to properly reform nuclear lamin filaments after cell division, and/or the loss of meshwork integrity, leads to the detachment of peripheral heterochromatin from the nuclear periphery and an extensive restructuring of chromatin domains. The molecular trigger for these changes may be the reduction of lamin B levels, which results in regions of the lamina being highly enriched in A-type lamins [14]. These appear to compromise the meshwork and generate nuclear blebs [14], the outward distortions of the nuclear envelope that are often observed in senescent cells. This nuclear blebbing triggers an innate immune response through the cGAS-STING pathway and promotes the SASP in both OIS and RS [15, 16] (for review see [12]). The disturbance of lamin-associated chromatin domains (LADs) was also found in cells from Down’s syndrome patients, where the 3D genome reorganization was tightly correlated with hallmarks of senescence [17]. This is consistent with other results showing that LADs contribute significantly to the functional organization of the nucleus [18–21].

During senescence in OIS cells, there is a loss of both facultative heterochromatin and constitutive LADs from the nuclear periphery leading in some cases to repeat expression, and resulting in an enormous reshaping of genome organization [22, 23]. Lamin B1 down-regulation is necessary but not sufficient for the formation of internal senescence-associated heterochromatin foci (SAHF) [24–27] (Fig. 2A-ii). The expression of the rest of the lamins is mostly unaffected, although there is often an accumulation of lamin A and pre-lamin A precursors [27–29]. At the same time as these lamina changes, there is an increase in nuclear pore density within the nuclear envelope, which appears to drive SAHF formation and SASP expression, possibly by excluding heterochromatin from the nuclear periphery [30]. In OIS cells, SAHF formation thus appears to result from a reorganization of pre-existing heterochromatin regions rather than from a de novo formation of new heterochromatin [31].

Fluorescence microscopy suggests that the dense internal SAHF structures result from heterochromatin-driven interactions between LADs, which bear either histone H3K27me3, H3K9me3 or both modifications, which are both hallmarks heterochromatin [22]. In OIS, SAHF were described as distinct, DNA-dense foci, enriched for heterochromatic markers such as H3K9me3, heterochromatin protein 1 (HP1), histone macroH2A, and linker histone H1 [24]. Now it is known that these chromatin foci have a multi-layered architecture, with a core of the constitutive H3K9me3 mark, and an outer ring carrying H3K27me3, the Polycomb modification [31, 32] that similar to replication timing resolved by super-resolution imaging [33]. The core and ring are surrounded by active chromatin, typically bearing H3K36me3 [32] (Fig. 2-iii). The segregation of H3K27me3 and H3K36me3 has been observed in many organisms, thus their separation is not senescence specific. This internal aggregation of H3K9me3-marked chromatin characterizes SAHF in OIS cells triggered by various inducers. However, the depletion of high-mobility group box 2 (HMGB2), a highly abundant chromatin-associated protein, is considered a hallmark for RS senescence entry in human cell lines [34, 35]. HMGB2 depletion also induces a dramatic reorganization of chromatin and the formation of senescence-induced CTCF clusters, which have no superposition with HP1α foci. These clusters are, therefore, distinct from SAHFs and are a hallmark of replicative senescence [34] (Fig. 2B).

With the advent of chromatin conformation capture methods (sequencing-based “C”-methods; henceforth called Hi-C) [36], our knowledge of genome structure has been revolutionized, allowing us to identify a hierarchy of structural units, the most prominent being DNA loops, topologically associated domains (TADs), A and B compartments (transcriptional active vs. inactive domains), and, on a larger scale, chromosome territories [37]. The first chromatin organization study of OIS cells using Hi-C analysis showed a loss of lamin-specific heterochromatic interactions, and a gain in short-range contacts within nuclear space, resembling the SAHF formation seen by microscopy [22]. Hi-C analysis of late replicative senescence (RS) similarly revealed an increase in short-range contacts and a decrease in long-range contacts, along with the switching of sub-groups of TADs compartments and conserved TAD boundaries [38]. However, higher resolution Hi-C in an early RS stage showed an increase in long-range interactions and only partial compartment switching [34]. These studies can be reconciled by suggesting a distinct evolution of the global organization of heterochromatin as SAHF mature, and with a higher proportion of regions switching from B to A compartments than from A to B [22, 38–40]. Depending on the senescence stage and stimulus used [39], heterochromatin showed distinct conformational changes, which almost surely implicates a role for chromatin remodelers.

In conclusion, the nuclear changes that typify all forms of cellular senescence are lamin cleavage, increased nuclear envelope permeability, disruption of the transport between the nucleus and cytoplasm, and changes in gene expression, along with changes in chromatin packing and architecture. Using machine learning to evaluate the interdependence of such phenotypes may allow one to use chromatin features to define cellular senescence states.

## Transcriptional programs of senescence affected by changes in chromatin organization

The dramatic changes in nuclear architecture and the remodeling of chromatin states during cellular senescence coincide with changes in transcriptional programs [41], as seen, for example, in the down-regulation of cell cycle genes in RS and their shift to a repressive compartment [42]. Other genes, such as those encoding the SASP secreted factors, show upregulation [43, 44]. Finding senescence-related gene expression signatures has been the focus of several global transcriptomic analyses [45–47]. Single-cell analyses showed that a senescent cell population comprises a range of cells with different mRNA expression profiles [46]. Consistently, transcriptome analysis of senescence induced by different stimuli showed vastly heterogeneous gene expression profiles and chromatin dynamics, which were also cell type dependent [47]. Nonetheless, 55 core genes could be identified as being associated with all senescent types and are defined as a senescence signature [47]. In this global analysis of senescence-associated gene expression, loci implicated in DNA replication, mitosis, and cell cycle are repressed, as they are when cells stop dividing due to RS or the excessive division [43, 48]. Besides the down-regulation of cell cycle genes, senescent cells are programmed to upregulate several chemokines, pro-inflammatory cytokines, and the matrix-remodeling enzymes that define the SASP [49, 50]. SASP is characteristic of all types of senescence, but how it is regulated depends on the cell type analysed, as well as the amount and time of exposure to the factor inducing senescence.

These dynamic characteristics of senescence gene expression are of course linked to chromatin states [51]. Given that SASP genes shift from a repressed to active state, the initial opening of chromatin is likely to require the action of pioneer transcription factors (TFs), a special class of factors that induce the opening of nucleosome-occupied chromatin structures to allow other TFs and cofactors to bind [52]. Recently, time-resolved genome-wide profile analysis identified the pioneer factor Activator Protein 1 (AP-1) as the trigger that activates the TF network driving the transcriptional program of OIS models [51]. AP-1 both "opens" the chromatin landscape of enhancers and is critical for the expression of SASP genes. The opening process starts with TF scanning, which entails the sliding of TFs along with DNA [53], and modulation of chromatin structure, possibly through the recruitment of histone modifiers or remodelers [54, 55]. This creates a permissive state for gene regulation [56, 57]. In addition, genome structures may facilitate transcription by forming loops that bring TF-bound enhancers close to promoters of target genes [58, 59]. While the relationship between 3D genome organization and TF activity [60] is well-studied in general, how the pioneer TF either shapes or is influenced by the 3D genome conformation during the fate transitions of senescence, is still unknown.

Abundant chromatin-associated proteins such as HMGA and HMGB families were also shown to play a role in the transcriptional changes necessary for senescence establishment [34, 35, 61]. In OIS, the overexpression of HMGA2 and collaboration with the p16^INK4a^ tumor suppressor promotes SAHF formation and stabilizes senescence [61]. In RS, on the other hand, other HMG proteins HMGB1 and HMGB2 are not associated with SAHF formation, but were shown to be linked to the expression of SASP genes [34, 35]. In particular, HMGB1 contributes to the coordinated activation of SASP by driving higher-order chromatin boundary changes upon RS entry [35]. Studying the mechanistic interaction of transcriptional activators, high-mobility group box proteins, and genome conformation during senescence will increase our understanding of both the epigenetic dynamics and the regulatory networks that govern senescence phenotypes.

## Mechanisms of chromatin reorganization in senescence

The striking changes in heterochromatin that occur during senescence have made SAHF a key model for investigating the link between chromatin reorganization and cell-fate transition. It remains unclear, however, if the changes that lead to SAHF formation imply two or more steps. A simple two-step mechanism was initially proposed for SAHF formation: the first step entailing heterochromatin segregation, and the second the spatial clustering of segregated regions [62] (Fig. 2A, B). This mechanism, built on the idea that SAHF formation is a static endpoint, is simple, yet it is now recognized that SAHF formation and maintenance are actually dynamic processes [51, 63]. There may be changes in structural and biophysical parameters that correlate with the reorganization of heterochromatin into SAHF and their maintenance, which require intermediate step(s) and other mechanisms, such as phase separation, viscoelasticity, and droplet nucleation [64, 65].

A 3D polymer model was introduced to capture chromatin organization in OIS cells, with a special focus on heterochromatin-lamina interaction [66]. This chromatin-lamina interaction was explained as desorbed phases due to the weak contacts, while heterochromatin-heterochromatin interaction led to (micro)phase separation of euchromatic and heterochromatic compartments. A quantitative two-parameter polymer model was proposed to explain the 3D genome reorganization of SAHF formation in OIS [39]. This modeling argued that the unleashing of heterochromatic domains from the lamina was enough to shape heterochromatin into SAHF, through micro-phase separation [39].

While these models have described the changes in the chromatin organization at compartments and TADs levels, a recent study examined how chromatin loops alter in OIS [67]. As a result of loop formation, OIS exhibits altered enhancer-promoter interactions, particularly at the Interleukin-1 (IL-1) cluster, which govern crucial SASP and cell cycle-related genes. Consequently, changes in enhancer-promoter contacts can be characteristic to transcription-dependent cohesin repositioning, by forming cohesin islands at the ends of active genes [68]. This results in new cohesin peaks and new loops. Since these models rely on data from fixed and ensemble Hi-C, the models do not address the heterogeneity introduced through chromatin dynamics and organization, most notably the formation and separation of H3K9me3 from H3K27me3 domains. To define the mechanisms underlying this organization, a more complete knowledge of the system derived from high resolution 4D microscopy (3D space over time) of chromatin states will be necessary, extending as well to changes that accompany the exit from the senescent state.

## Senescence exit

Senescence was originally thought to be an irreversible cell state, yet now various studies have shown that it is reversible: cells can exit senescence either by manipulating the key effectors of senescence, such as p53, p16, H3K9me3, or by changing the senescence-associated transcriptional program [69–71]. In replicative senescence, low expression levels of p16 allowed human senescent cells to resume growth upon the inactivation of p53 [72]. Likewise, the suppression of p53 in senescent mouse embryo fibroblasts led to the loss of senescence-related gene expression, a reversal of senescence and rapid re-entry into the cell cycle [73]. A study conducted in vivo revealed that genetically switchable models of senescence targeting p53 or H3K9me3, were able to bypass senescence, allowing cells to enter the cell cycle while displaying stem cell characteristics [74].

Acting upstream of these effector molecules, it was shown that expression of the Yamanaka factors, Oct4, Sox2, Klf4, and c-Myc, can reactivate cell proliferation and partially reverse the senescence phenotypes [69]. In another study, it was shown that the depletion of the pioneer factor AP-1 partially reverts the senescence transcriptional program, decreasing the expression of SASP genes [51]. Although this study highlighted the role of epigenetic remodeling as driving senescence exit, the molecular mechanism(s) explaining this process are unclear. Senescence exit could also be triggered by depleting the long noncoding RNA called PANDA, for P21-associated ncRNA DNA damage-activated [70], which limits the expression of NF-YA-E2F-coregulated proliferation-promoting genes. Very recently, senescence exit into a quiescent state without proliferation was shown to be prompted by the inhibition of a 3-phosphoinositide–dependent protein kinase 1 (PDK1) [71], loss of which suppresses both NFκB and mTOR signaling by inhibiting a growth stimulatory loop of PDK1, AKT, IDBKB, and PTEN [71]. This leads to considering the inhibition of PDK1 as a promising target for the senescent exit.

Whereas molecular transcriptomics and fixed cell imaging have implicated these regulatory players in the exit from senescence, they have not been able to link genome reorganization to the altered gene expression patterns. It remains to be seen how chromatin reorganizes globally with respect to the transcriptome during both entry into and exit from senescence.

## Future directions

Senescence is a dynamic process that includes an early cell cycle arrest, a period of chromatin remodeling, the opening and reorganization of heterochromatin, and finally a well-defined set of transcriptional changes. Transcriptomic studies have confirmed that senescence is not a single distinct state, thus it remains a challenge to find robust markers for this range of senescent states. The advent of advanced, next-generation profiling tools, such as single-cell RNA sequencing, has accelerated our understanding of the heterogeneity of senescence [75, 76]. The use of artificial intelligence combined with nuclear-imaging techniques is needed to characterize senescence phenotypes, and to enable pathologists to definitively identify cells as senescent [77].

It is clear that chromatin reorganization is a major player in senescence, yet it is unclear which mechanisms trigger the changes that are crucial either at senescence entry or exit. Global changes in chromatin vary depending on the stressor used, the time of induction and the cell type. These differences suggest that SAHF may also form through somewhat different mechanisms, depending on the stressor used. If so, it will be important to identify both the essential commonality underlying SAHF formation, and the stress-specific aspects. To this end, time-resolved, high resolution measurements of changes in 3D chromatin dynamics must be correlated with changes in transcriptional output during the induction of senescence by different stressors in single living cells [78–81]. This would allow one to investigate both global and local changes in chromatin mobility over specific genomic regions (e.g., SAHF vs heterochromatin vs euchromatin).

Addressing these questions may allow us to distinguish among the hypotheses put forth to explain heterochromatin reorganization, namely, activity-driven microphase separation [27, 28], or the involvement of switchable bridging proteins (with switching rates that control SAHF size) [29]. Potential intermediate steps with distinct structural properties may exist, which could shed light on new mechanisms involved in heterochromatin formation, whether during normal cell differentiation or in senescence. Both characterizing early markers of nuclear rearrangement and SAHF formation, and being able to interfere with them, will be relevant to the treatment of cancer and age-related disorders.

It is clear that 3D genome organization reflects TF activity, most obvious being the pairing of TF-bound enhancers to the appropriate proximal promotor at a target gene [59, 82, 83]. Proximity to a chromatin compartment, as well as B-to-A compartment switches, enable super-enhancer function and the coordinated expression of sets of genes [84]. The link between enhancer-promoter long-range interaction to transcriptional output has been demonstrated in situ by the combination of multiplexed super-resolution imaging and RNA FISH in living single-cells [85]. This combination has revealed domain structures that change with cell identity. With imaging-based transcriptomics, it will be possible to image up to ~10,000 different RNA species in a single cell [86, 87], and to identify cell cycle-independent spatial heterogeneity of distinct cells, based on gene expression patterns [87]. This may provide a means to quantify both expression and spatial information of RNAs in individual cells within tissue environments [86].

The study of senescence underscores the need to map how the 3D dynamics of chromatin structure is affected by changes in the cellular transcriptome, and vice versa. Coupling the new single-nuclei imaging tools with spatially resolved single-cell transcriptomics methods will open new channels for linking the dynamic aspects of chromatin structure to transcriptional changes [88]. In addition, identifying the biophysical principles that govern these dynamic changes in structure will give us a handle of the upstream mechanisms that control chromatin dynamics and their concurrent epigenetic changes during senescence entry and exit. New analytical methods allow one to assess the dynamic and (bio)physical properties of chromatin as it undergoes structural changes and to couple this with transcriptional states [71–73]. This will reveal the causal relationship between inducors [89] and the genome (re-)organization that accompany transcriptional control. Understanding senescence on this level has the added value of allowing one to develop markers relevant for disease diagnostics and therapeutic intervention.

### Reporting summary

Further information on research design is available in the Nature Research Reporting Summary linked to this article.

## Supplementary information

Reporting Summary

## References

[1] Hayflick L, Moorhead PS. The serial cultivation of human diploid cell strains. Exp Cell Res. 1961. 10.1016/0014-4827(61)90192-6. 10.1016/0014-4827(61)90192-6 13905658

[2] Hernandez-Segura A, Nehme J, Demaria M. Hallmarks of cellular senescence. Trends Cell Biol. 2018. 10.1016/j.tcb.2018.02.001. 10.1016/j.tcb.2018.02.001 29477613

[3] Coppé JP, Desprez PY, Krtolica A, Campisi J. The senescence-associated secretory phenotype: the dark side of tumor suppression. Annu Rev Pathol Mech Dis. 2010. 10.1146/annurev-pathol-121808-102144. 10.1146/annurev-pathol-121808-102144 PMC4166495 20078217

[4] Childs BG, Baker DJ, Kirkland JL, Campisi J, van Deursen JM. Senescence and apoptosis: dueling or complementary cell fates? EMBO Rep. 2014;15:1139–53. 25312810 10.15252/embr.201439245 PMC4253488

[5] Campisi J, D’Adda Di Fagagna F. Cellular senescence: when bad things happen to good cells. Nat Rev Mol Cell Biol. 2007. 10.1038/nrm2233. 10.1038/nrm2233 17667954

[6] Acosta JC, Banito A, Wuestefeld T, Georgilis A, Janich P, Morton JP, et al. A complex secretory program orchestrated by the inflammasome controls paracrine senescence. Nat Cell Biol. 2013. 10.1038/ncb2784. 10.1038/ncb2784 PMC3732483 23770676

[7] Hayflick L. The limited in vitro lifetime of human diploid cell strains. Exp Cell Res. 1965. 10.1016/0014-4827(65)90211-9. 10.1016/0014-4827(65)90211-9 14315085

[8] Serrano M, Lin AW, McCurrach ME, Beach D, Lowe SW. Oncogenic ras provokes premature cell senescence associated with accumulation of p53 and p16(INK4a). Cell. 1997;88:593–602. 10.1016/S0092-8674(00)81902-9. 9054499 10.1016/s0092-8674(00)81902-9

[9] Fridlyanskaya I, Alekseenko L, Nikolsky N. Senescence as a general cellular response to stress: a mini-review. Exp Gerontol. 2015. 10.1016/j.exger.2015.09.021. 10.1016/j.exger.2015.09.021 26435346

[10] Fan DNY, Schmitt CA. Genotoxic stress-induced senescence. Methods Mol Biol. 2019. 10.1007/978-1-4939-8931-7_10. 10.1007/978-1-4939-8931-7_10 30474843

[11] Hao X, Wang C, Zhang R. Chromatin basis of the senescence-associated secretory phenotype. Trends Cell Biol. 2022. 10.1016/j.tcb.2021.12.003. 10.1016/j.tcb.2021.12.003 PMC9106822 35012849

[12] Rocha A, Dalgarno A, Neretti N. The functional impact of nuclear reorganization in cellular senescence. Brief Funct Genom. 2021. 10.1093/bfgp/elab012. 10.1093/bfgp/elab012 PMC8789270 33755107

[13] Evans SA, Horrell J, Neretti N. The three-dimensional organization of the genome in cellular senescence and age-associated diseases. Semin Cell Dev Biol. 2019. 10.1016/j.semcdb.2018.07.022. 10.1016/j.semcdb.2018.07.022 PMC6661233 30031215

[14] Funkhouser CM, Sknepnek R, Shimi T, Goldman AE, Goldman RD, De La Cruz MO. Mechanical model of blebbing in nuclear lamin meshworks. Proc Natl Acad Sci USA. 2013;110:3248–53. 23401537 10.1073/pnas.1300215110 PMC3587257

[15] Dou Z, Ghosh K, Vizioli MG, Zhu J, Sen P, Wangensteen KJ, et al. Cytoplasmic chromatin triggers inflammation in senescence and cancer. Nature. 2017;550:402–6. 28976970 10.1038/nature24050 PMC5850938

[16] Glück S, Guey B, Gulen MF, Wolter K, Kang T-W, Schmacke NA, et al. Innate immune sensing of cytosolic chromatin fragments through cGAS promotes senescence. Nat Cell Biol. 2017;19:1061–70. 28759028 10.1038/ncb3586 PMC5826565

[17] Meharena HS, Marco A, Dileep V, Lockshin ER, Akatsu GY, Mullahoo J, et al. Down-syndrome-induced senescence disrupts the nuclear architecture of neural progenitors. Cell Stem Cell. 2022;29:116–130.e7. 34995493 10.1016/j.stem.2021.12.002 PMC8805993

[18] Briand N, Collas P. Lamina-associated domains: peripheral matters and internal affairs. Genome Biol. 2020. 10.1186/s13059-020-02003-5. 10.1186/s13059-020-02003-5 PMC7114793 32241294

[19] Zheng X, Hu J, Yue S, Kristiani L, Kim M, Sauria M, et al. Lamins organize the global three-dimensional genome from the nuclear periphery. Mol Cell. 2018. 10.1016/j.molcel.2018.05.017. 10.1016/j.molcel.2018.05.017 PMC6886264 30201095

[20] Brueckner L, Zhao PA, van Schaik T, Leemans C, Sima J, Peric‐Hupkes D, et al. Local rewiring of genome–nuclear lamina interactions by transcription. EMBO J. 2020. 10.15252/embj.2019103159. 10.15252/embj.2019103159 PMC7073462 32080885

[21] Guerreiro I, Kind J. Spatial chromatin organization and gene regulation at the nuclear lamina. Curr Opin Genet Dev. 2019. 10.1016/j.gde.2019.04.008. 10.1016/j.gde.2019.04.008 PMC7100903 31112905

[22] Chandra T, Ewels PA, Schoenfelder S, Furlan-Magaril M, Wingett SW, Kirschner K, et al. Global reorganization of the nuclear landscape in senescent cells. Cell Rep. 2015. 10.1016/j.celrep.2014.12.055. 10.1016/j.celrep.2014.12.055 PMC4542308 25640177

[23] Lenain C, De Graaf CA, Pagie L, Visser NL, De Haas M, De Vries SS, et al. Massive reshaping of genome-nuclear lamina interactions during oncogene-induced senescence. Genome Res. 2017. 10.1101/gr.225763.117. 10.1101/gr.225763.117 PMC5630027 28916540

[24] Narita M, Nũnez S, Heard E, Narita M, Lin AW, Hearn SA, et al. Rb-mediated heterochromatin formation and silencing of E2F target genes during cellular senescence. Cell. 2003. 10.1016/S0092-8674(03)00401-X. 10.1016/s0092-8674(03)00401-x 12809602

[25] Zhang R, Chen W, Adams PD. Molecular dissection of formation of senescence-associated heterochromatin foci. Mol Cell Biol. 2007. 10.1128/mcb.02019-06. 10.1128/MCB.02019-06 PMC1820509 17242207

[26] Zampetidis CP, Galanos P, Angelopoulou A, Zhu Y, Polyzou A, Karamitros T, et al. A recurrent chromosomal inversion suffices for driving escape from oncogene-induced senescence via subTAD reorganization. Mol Cell. 2021. 10.1016/j.molcel.2021.10.017. 10.1016/j.molcel.2021.10.017 34793711

[27] Sadaie M, Salama R, Carroll T, Tomimatsu K, Chandra T, Young ARJ, et al. Redistribution of the lamin B1 genomic binding profile affects rearrangement of heterochromatic domains and SAHF formation during senescence. Genes Dev. 2013. 10.1101/gad.217281.113. 10.1101/gad.217281.113 PMC3759696 23964094

[28] Shimi T, Butin-Israeli V, Adam SA, Hamanaka RB, Goldman AE, Lucas CA, et al. The role of nuclear lamin B1 in cell proliferation and senescence. Genes Dev. 2011. 10.1101/gad.179515.111. 10.1101/gad.179515.111 PMC3248680 22155925

[29] Ukekawa R, Miki K, Fujii M, Hirano H, Ayusawa D. Accumulation of multiple forms of lamin A with down-regulation of FACE-1 suppresses growth in senescent human cells. Genes Cells. 2007. 10.1111/j.1365-2443.2007.01057.x. 10.1111/j.1365-2443.2007.01057.x 17352743

[30] Boumendil C, Hari P, Olsen KCF, Acosta JC, Bickmore WA. Nuclear pore density controls heterochromatin reorganization during senescence. Genes Dev. 2019. 10.1101/gad.321117.118. 10.1101/gad.321117.118 PMC6362808 30692205

[31] Chandra T, Kirschner K. Chromosome organisation during ageing and senescence. Curr Opin Cell Biol. 2016. 10.1016/j.ceb.2016.03.020. 10.1016/j.ceb.2016.03.020 27101466

[32] Chandra T, Kirschner K, Thuret JY, Pope BD, Ryba T, Newman S, et al. Independence of repressive histone marks and chromatin compaction during senescent heterochromatic layer formation. Mol Cell. 2012. 10.1016/j.molcel.2012.06.010. 10.1016/j.molcel.2012.06.010 PMC3701408 22795131

[33] Miron E, Oldenkamp R, Brown JM, Pinto DMS, Xu CS, Faria AR, et al. Chromatin arranges in chains of mesoscale domains with nanoscale functional topography independent of cohesin. Sci Adv. 2020. 10.1126/sciadv.aba8811. 10.1126/sciadv.aba8811 PMC7531892 32967822

[34] Zirkel A, Nikolic M, Sofiadis K, Mallm JP, Brackley CA, Gothe H, et al. HMGB2 Loss upon senescence entry disrupts genomic organization and induces CTCF clustering across cell types. Mol Cell. 2018. 10.1016/j.molcel.2018.03.030. 10.1016/j.molcel.2018.03.030 29706538

[35] Sofiadis K, Josipovic N, Nikolic M, Kargapolova Y, Varamogianni-mamatsi V, Zirkel A, et al. HMGB 1 coordinates SASP-related chromatin folding and RNA homeostasis on the path to senescence. Mol Syst Biol. 2021;17:e9760. 10.15252/msb.20209760 PMC8224457 34166567

[36] Dekker J, Rippe K, Dekker M, Kleckner N. Capturing chromosome conformation. Science. 2002;295:1306–11. 11847345 10.1126/science.1067799

[37] Fortin JP, Hansen KD. Reconstructing A/B compartments as revealed by Hi-C using long-range correlations in epigenetic data. Genome Biol. 2015. 10.1186/s13059-015-0741-y. 10.1186/s13059-015-0741-y PMC4574526 26316348

[38] Criscione SW, Cecco M De, Siranosian B, Zhang Y, Kreiling JA, Sedivy JM, et al. Biomolecules: reorganization of chromosome architecture in replicative cellular senescence. Sci Adv. 2016. 10.1126/sciadv.1500882. 10.1126/sciadv.1500882 PMC4788486 26989773

[39] Sati S, Bonev B, Szabo Q, Jost D, Bensadoun P, Serra F, et al. 4D genome rewiring during oncogene-induced and replicative senescence. Mol Cell. 2020. 10.1016/j.molcel.2020.03.007. 10.1016/j.molcel.2020.03.007 PMC7208559 32220303

[40] Iwasaki O, Tanizawa H, Kim KD, Kossenkov A, Nacarelli T, Tashiro S, et al. Involvement of condensin in cellular senescence through gene regulation and compartmental reorganization. Nat Commun. 2019. 10.1038/s41467-019-13604-5. 10.1038/s41467-019-13604-5 PMC6908677 31831736

[41] Criscione SW, Teo YV, Neretti N. The Chromatin landscape of cellular senescence. Trends Genet. 2016. 10.1016/j.tig.2016.09.005. 10.1016/j.tig.2016.09.005 PMC5235059 27692431

[42] Tomimatsu K, Bihary D, Olan I, Parry AJ, Schoenfelder S, Chan ASL, et al. Locus-specific induction of gene expression from heterochromatin loci during cellular senescence. Nat Aging. 2021. 10.1038/s43587-021-00147-y. 10.1038/s43587-021-00147-y 37118356

[43] Zhang H, Pan KH, Cohen, SN. Senescence-specific gene expression fingerprints reveal cell-type-dependent physical clustering of up-regulated chromosomal loci. Proc Natl Acad Sci USA. 2003. 10.1073/pnas.2627983100. 10.1073/pnas.2627983100 PMC152278 12626749

[44] Saul D, Kosinsky RL, Atkinson EJ, Doolittle ML, Zhang X, LeBrasseur NK, et al. A new gene set identifies senescent cells and predicts senescence-associated pathways across tissues. Nat Commun. 2022. 10.1038/s41467-022-32552-1. 10.1038/s41467-022-32552-1 PMC9381717 35974106

[45] Casella G, Munk R, Kim KM, Piao Y, De S, Abdelmohsen K, et al. Transcriptome signature of cellular senescence. Nucleic Acids Res. 2019. 10.1093/nar/gkz555. 10.1093/nar/gkz555 PMC6698740 31251810

[46] Wiley CD, Flynn JM, Morrissey C, Lebofsky R, Shuga J, Dong X, et al. Analysis of individual cells identifies cell-to-cell variability following induction of cellular senescence. Aging Cell. 2017. 10.1111/acel.12632. 10.1111/acel.12632 PMC5595671 28699239

[47] Hernandez-Segura A, de Jong TV, Melov S, Guryev V, Campisi J, Demaria M. Unmasking transcriptional heterogeneity in senescent cells. Curr Biol. 2017. 10.1016/j.cub.2017.07.033. 10.1016/j.cub.2017.07.033 PMC5788810 28844647

[48] Kim YM, Byun HO, Jee BA, Cho H, Seo YH, Kim YS, et al. Implications of time-series gene expression profiles of replicative senescence. Aging Cell. 2013. 10.1111/acel.12087. 10.1111/acel.12087 23590226

[49] Coppé JP, Patil CK, Rodier F, Sun Y, Muñoz DP, Goldstein J, et al. Senescence-associated secretory phenotypes reveal cell-nonautonomous functions of oncogenic RAS and the p53 tumor suppressor. PLoS Biol. 2008. 10.1371/journal.pbio.0060301. 10.1371/journal.pbio.0060301 PMC2592359 19053174

[50] Rodier F, Coppé JP, Patil CK, Hoeijmakers WAM, Muñoz DP, Raza SR, et al. Persistent DNA damage signalling triggers senescence-associated inflammatory cytokine secretion. Nat Cell Biol. 2009. 10.1038/ncb1909. 10.1038/ncb1909 PMC2743561 19597488

[51] Martínez-Zamudio RI, Roux PF, de Freitas JANLF, Robinson L, Doré G, Sun B, et al. AP-1 imprints a reversible transcriptional programme of senescent cells. Nat Cell Biol. 2020. 10.1038/s41556-020-0529-5. 10.1038/s41556-020-0529-5 PMC7899185 32514071

[52] Soufi A, Garcia MF, Jaroszewicz A, Osman N, Pellegrini M, Zaret KS. Pioneer transcription factors target partial DNA motifs on nucleosomes to initiate reprogramming. Cell. 2015. 10.1016/j.cell.2015.03.017. 10.1016/j.cell.2015.03.017 PMC4409934 25892221

[53] Hettich J, Gebhardt JCM. Transcription factor target site search and gene regulation in a background of unspecific binding sites. J Theor Biol. 2018. 10.1016/j.jtbi.2018.05.037. 10.1016/j.jtbi.2018.05.037 PMC6103292 29870697

[54] Cagnetta F, Michieletto D, Marenduzzo D. Nonequilibrium strategy for fast target search on the genome. Phys Rev Lett. 2020. 10.1103/PhysRevLett.124.198101. 10.1103/PhysRevLett.124.198101 32469558

[55] Xin B, Rohs R. Relationship between histone modifications and transcription factor binding is protein family specific. Genome Res. 2018. 10.1101/gr.220079.116. 10.1101/gr.220079.116 PMC5848611 29326300

[56] Zaret KS, Carroll JS. Pioneer transcription factors: establishing competence for gene expression. Genes Dev. 2011. 10.1101/gad.176826.111. 10.1101/gad.176826.111 PMC3219227 22056668

[57] Iwafuchi-Doi M, Zaret KS. Cell fate control by pioneer transcription factors. Development. 2016. 10.1242/dev.133900. 10.1242/dev.133900 PMC6514407 27246709

[58] Furlong EEM, Levine M. Developmental enhancers and chromosome topology. Science. 2018. 10.1126/science.aau0320. 10.1126/science.aau0320 PMC6986801 30262496

[59] Kim S, Shendure J. Mechanisms of interplay between transcription factors and the 3D genome. Mol Cell. 2019. 10.1016/j.molcel.2019.08.010. 10.1016/j.molcel.2019.08.010 31521504

[60] Shaban HA, Suter DM. Individual activator and repressor transcription factors induce global changes in chromatin mobility. bioRxiv. 2022. 10.1101/2022.04.12.488001.

[61] Narita M, Narita M, Krizhanovsky V, Nuñez S, Chicas A, Hearn SA, et al. A Novel role for high-mobility group A proteins in cellular senescence and heterochromatin formation. Cell. 2006;126:503–14. 16901784 10.1016/j.cell.2006.05.052

[62] Chandra T. Senescence associated heterochromatic foci: SAHF. In: Bazett-Jones, D., Dellaire, G. (eds) The functional nucleus. Cham: Springer; 2016. 10.1007/978-3-319-38882-3_9.

[63] Van Deursen JM. The role of senescent cells in ageing. Nature. 2014. 10.1038/nature13193. 10.1038/nature13193 PMC4214092 24848057

[64] Erdel F, Rippe K. Formation of chromatin subcompartments by phase separation. Biophys J. 2018;114:2262–70. 29628210 10.1016/j.bpj.2018.03.011 PMC6129460

[65] Michieletto D, Marenda M. Rheology and viscoelasticity of proteins and nucleic acids condensates. JACS Au. 2022. 10.1021/jacsau.2c00055. 10.1021/jacsau.2c00055 PMC9326828 35911447

[66] Chiang M, Michieletto D, Brackley CA, Rattanavirotkul N, Mohammed H, Marenduzzo D, et al. Polymer modeling predicts chromosome reorganization in senescence. Cell Rep. 2019. 10.1016/j.celrep.2019.08.045. 10.1016/j.celrep.2019.08.045 PMC6859504 31533042

[67] Olan I, Parry AJ, Schoenfelder S, Narita M, Ito Y, Chan ASL, et al. Transcription-dependent cohesin repositioning rewires chromatin loops in cellular senescence. Nat Commun. 2020. 10.1038/s41467-020-19878-4. 10.1038/s41467-020-19878-4 PMC7695716 33247104

[68] Busslinger GA, Stocsits RR, Van Der Lelij P, Axelsson E, Tedeschi A, Galjart N, et al. Cohesin is positioned in mammalian genomes by transcription, CTCF and Wapl. Nature. 2017. 10.1038/nature22063. 10.1038/nature22063 PMC6080695 28424523

[69] Ocampo A, Reddy P, Martinez-Redondo P, Platero-Luengo A, Hatanaka F, Hishida T, et al. In vivo amelioration of age-associated hallmarks by partial reprogramming. Cell. 2016. 10.1016/j.cell.2016.11.052. 10.1016/j.cell.2016.11.052 PMC5679279 27984723

[70] Puvvula PK, Desetty RD, Pineau P, Marchio A, Moon A, Dejean A, et al. Long noncoding RNA PANDA and scaffold-attachment-factor SAFA control senescence entry and exit. Nat Commun. 2014. 10.1038/ncomms6323. 10.1038/ncomms6323 PMC4263151 25406515

[71] An S, Cho SY, Kang J, Lee S, Kim HS, Min DJ, et al. Inhibition of 3-phosphoinositide-dependent protein kinase 1 (PDK1) can revert cellular senescence in human dermal fibroblasts. Proc Natl Acad Sci USA. 2020. 10.1073/pnas.1920338117. 10.1073/pnas.1920338117 PMC7733858 33229519

[72] Beauséjour CM, Krtolica A, Galimi F, Narita M, Lowe SW, Yaswen P, et al. Reversal of human cellular senescence: roles of the p53 and p16 pathways. EMBO J 2003. 10.1093/emboj/cdg417. 10.1093/emboj/cdg417 PMC175806 12912919

[73] Dirac AMG, Bernards R. Reversal of senescence in mouse fibroblasts through lentiviral suppression of p53. J Biol Chem. 2003;278:11731–4. 12551891 10.1074/jbc.C300023200

[74] Milanovic M, Fan DNY, Belenki D, Däbritz JHM, Zhao Z, Yu Y, et al. Senescence-associated reprogramming promotes cancer stemness. Nature. 2018. 10.1038/nature25167. 10.1038/nature25167 29258294

[75] Hughes BK, Wallis R, Bishop CL. Yearning for machine learning: applications for the classification and characterisation of senescence. Cell Tissue Res. 2023. 10.1007/s00441-023-03768-4. 10.1007/s00441-023-03768-4 PMC10558380 37016180

[76] Teo YV, Rattanavirotkul N, Olova N, Salzano A, Quintanilla A, Tarrats N, et al. Notch signaling mediates secondary senescence. Cell Rep. 2019. 10.1016/j.celrep.2019.03.104. 10.1016/j.celrep.2019.03.104 PMC6486482 31018144

[77] Heckenbach I, Mkrtchyan GV, Ezra MB, Bakula D, Madsen JS, Nielsen, MH et al. Nuclear morphology is a deep learning biomarker of cellular senescence. Nat Aging. 2022. 10.1038/s43587-022-00263-3. 10.1038/s43587-022-00263-3 PMC10154217 37118134

[78] Shaban HA, Barth R, Bystricky K. Formation of correlated chromatin domains at nanoscale dynamic resolution during transcription. Nucleic Acids Res. 2018;46:e77–e77. 29718294 10.1093/nar/gky269 PMC6061878

[79] Shaban HA, Barth R, Recoules L, Bystricky K. Hi-D: nanoscale mapping of nuclear dynamics in single living cells. Genome Biol. 2020;21:95. 32312289 10.1186/s13059-020-02002-6 PMC7168861

[80] Barth R, Bystricky K, Shaban HA. Coupling chromatin structure and dynamics by live super-resolution imaging. Sci Adv. 2020;6. 10.1126/sciadv.aaz2196. 10.1126/sciadv.aaz2196 PMC7458449 32937447

[81] Shaban HA, Seeber A. Monitoring the spatio-temporal organization and dynamics of the genome. Nucleic Acids Res. 2020. 10.1093/nar/gkaa135. 10.1093/nar/gkaa135 PMC7144944 32123910

[82] Hsieh THS, Cattoglio C, Slobodyanyuk E, Hansen AS, Rando OJ, Tjian R, et al. Resolving the 3D landscape of transcription-linked mammalian chromatin folding. Mol Cell. 2020;78:539–553.e8. 32213323 10.1016/j.molcel.2020.03.002 PMC7703524

[83] Kim S, Wysocka J. Deciphering the multi-scale, quantitative cis-regulatory code. Mol Cell. 2023;83:373–92. 36693380 10.1016/j.molcel.2022.12.032 PMC9898153

[84] Stadhouders R, Vidal E, Serra F, Di Stefano B, Le Dily F, Quilez J, et al. Transcription factors orchestrate dynamic interplay between genome topology and gene regulation during cell reprogramming. Nat Genet. 2018. 10.1038/s41588-017-0030-7. 10.1038/s41588-017-0030-7 PMC5810905 29335546

[85] Mateo LJ, Murphy SE, Hafner A, Cinquini IS, Walker CA, Boettiger AN. Visualizing DNA folding and RNA in embryos at single-cell resolution. Nature. 2019. 10.1038/s41586-019-1035-4. 10.1038/s41586-019-1035-4 PMC6556380 30886393

[86] Chen KH, Boettiger AN, Moffitt JR, Wang S, Zhuang X. Spatially resolved, highly multiplexed RNA profiling in single cells. Science. 2015;348. 10.1126/science.aaa6090. 10.1126/science.aaa6090 PMC4662681 25858977

[87] Xia C, Fan J, Emanuel G, Hao J, Zhuang X. Spatial transcriptome profiling by MERFISH reveals subcellular RNA compartmentalization and cell cycle-dependent gene expression. Proc Natl Acad Sci USA. 2019. 10.1073/pnas.1912459116. 10.1073/pnas.1912459116 PMC6765259 31501331

[88] Agbleke AA, Amitai A, Buenrostro JD, Chakrabarti A, Chu L, Hansen AS, et al. Advances in chromatin and chromosome research: perspectives from multiple fields. Mol Cell. 2020. 10.1016/j.molcel.2020.07.003. 10.1016/j.molcel.2020.07.003 PMC7888594 32768408

[89] Barth R, Fourel G, Shaban HA. Dynamics as a cause for the nanoscale organization of the genome. Nucleus. 2020;11:83–98. 32449444 10.1080/19491034.2020.1763093 PMC7529413

